# Education for a tobacco free world: the 11^th^ meeting of the International Society for the Prevention of Tobacco Induced Diseases (ISPTID)

**DOI:** 10.1186/1617-9625-12-S1-A1

**Published:** 2014-06-06

**Authors:** Constantine Vardavas, Charis Girvalaki, Christina Gratsiou, Panagiotis Behrakis

**Affiliations:** 1 Center for Global Tobacco Control, Department of Social and Behavioral Sciences, Harvard School of Public Health, Boston, USA; 2 Smoking and Lung Cancer Research Center, Hellenic Cancer Society, Athens, 11521, Greece; 3 Biomedical Research Foundation of the Academy of Athens, Athens, 115 27, Greece; 4 Department of Social Medicine, Clinic of Social and Family Medicine, University of Crete, Heraklion, Greece; 5 1st Respiratory Department, University of Athens, Medical School, Sotiria Hospital, Athens, 11527, Greece; 6 Department of Environmental Health, Harvard School of Public Health, 677 Huntington Avenue Landmark, West Boston, MA 02115, USA

## 

With tobacco the single most preventable cause of death and disability on the planet and potentially responsible for more than a billion deaths this century –if urgent action is not taken- the importance of tackling the tobacco epidemic at its roots is clearly apparent [1].

Within the context of tackling this public health behemoth, the 11^th^ meeting of the International Society for the Prevention of Tobacco Induced Diseases (ISPTID) in Athens, Greece took place in tandem with the 4^th^ Panhellenic Congress on Tobacco Control in December 2013. The conference brought together a multidisciplinary group of participants in two separate, but inter-complimentary, tracks: one focused on the scientific aspects of the prevention of tobacco attributable diseases, and one focused on education and learning for a tobacco free world.

## Track 1: the science behind preventing tobacco use

Over 250 participants from countries spanning the geography of the planet followed 62 oral/keynote presentations and 26 lively poster discussions within the auditorium of the Biomedical Research Foundation of the Academy of Athens (BRFAA). So as to limit participant dispersion between rooms, parallel sessions were limited to a maximum of two at a time. Scientific support was provided by the journal, Tobacco Induced Diseases –which also hosts the current abstract book of selected presentations. Topics covered multiple areas including: Modified risk products, e-cigarettes, tobacco use and disease epidemiology, occupational health, clinical impact of tobacco induced diseases, molecular/cellular impact of tobacco use, smoking cessation, and finally educational and community interventions for reducing tobacco use among youth. Within the context of the conference the ED Nelson award was also presented to the best first appearing young researcher in tobacco control.

## Track 2: education for a tobacco free future

Adolescence is a crucial period in human development, as during this phase the majority of smokers experiment with their first cigarettes, a risky behaviour which may continue into adulthood, that however can be prevented with school or community based strategic actions that enhance and facilitate the adoption of a tobacco free life [2]. School prevention programs which start in upper elementary or middle school, and continue into high school are necessary and able to provide important results towards rejecting smoking initiation [3]. Within the context of the joint 11th IISPTID meeting with the 4th Panhellenic Congress on Tobacco Control a special parallel track and specific day was designed so as to facilitate training of teachers and educators, and also peer to peer training between school students. This track was followed by a total of 1340 students, within the auditorium of the Ministry of Education of Greece, and 1041 online school connections, 69 of which followed the session via distance in real time. During the process leading up to this conference, students submitted more than a thousand drawings, works of art, and short videos as part of the national campaign for a tobacco free future, the best of which were awarded in the closing ceremony –which ended with a live POP concert for a tobacco free future [4].

In conclusion, the conference’s main take home was clear and simple: Education, whether peer to peer, or through the community or school system is an integral part of preventing tobacco induced diseases. Together a tobacco free future is possible and education can pave the way.

## Competing interests

The authors declare that they have no competing interests.

## Authors' contributions

All authors contributed equally in preparation of this manuscript. All authors read and approved the final manuscript.
